# An Exploratory Radiomics Approach to Quantifying Pulmonary Function in CT Images

**DOI:** 10.1038/s41598-019-48023-5

**Published:** 2019-08-08

**Authors:** Kyle J. Lafata, Zhennan Zhou, Jian-Guo Liu, Julian Hong, Chris R. Kelsey, Fang-Fang Yin

**Affiliations:** 1 Department of Radiation Oncology, Duke University Medical Center, Durham, NC USA; 2 Department of Physics, Duke University, Durham, NC USA; 3 Beijing International Center of Mathematical Research, Peking University, Beijing, China; 4 Department of Mathematics, Duke University, Durham, NC USA

**Keywords:** Diagnostic markers, Three-dimensional imaging, Applied mathematics, Computational science

## Abstract

Contemporary medical imaging is becoming increasingly more quantitative. The emerging field of radiomics is a leading example. By translating unstructured data (i.e., images) into structured data (i.e., imaging features), radiomics can potentially characterize clinically useful imaging phenotypes. In this paper, an exploratory radiomics approach is used to investigate the potential association between quantitative imaging features and pulmonary function in CT images. Thirty-nine radiomic features were extracted from the lungs of 64 patients as potential imaging biomarkers for pulmonary function. Collectively, these features capture the morphology of the lungs, as well as intensity variations, fine-texture, and coarse-texture of the pulmonary tissue. The extracted lung radiomics data was compared to conventional pulmonary function tests. In general, patients with larger lungs of homogeneous, low attenuating pulmonary tissue (as measured via radiomics) were found to be associated with poor spirometry performance and a lower diffusing capacity for carbon monoxide. Unsupervised dynamic data clustering revealed subsets of patients with similar lung radiomic patterns that were found to be associated with similar forced expiratory volume in one second (FEV_1_) measurements. This implies that patients with similar radiomic feature vectors also presented with comparable spirometry performance, and were separable by varying degrees of pulmonary function as measured by imaging.

## Introduction

The field of *radiomics* attempts to identify computational biomarkers potentially hidden within high-throughput imaging data^1^. To date, most radiomic applications have largely focused on the characterization of tumor phenotypes. In fact, several remarkable relationships have been identified that link radiomics data to tumor histology^2,3^, genomic underpinnings^4^, and treatment response^5–11^. However, fewer studies have been designed for non-tumor radiomic analysis. This paper investigates the association between pulmonary function and radiomic features extracted from the lungs of computed tomography (CT) images. It is generally well-known that changes in lung function can be mechanistically explained based on morphological CT features (e.g., bronchial thickening, honeycombing, etc.). Nevertheless, such phenomena are typically based on expert observation and are therefore often difficult to quantify. A quantitative radiomics approach has the potential to objectively measure these observations, and may eventually serve as a clinically useful tool that is complimentary to the expert radiologist.

The objective of this work was to investigate the correlation of radiomic features extracted from the lungs on CT images with two common pulmonary function tests (PFTs): (1) *forced expiratory volume in one second* (*FEV*_1_) measurements and (2) *diffusing capacity of the lung for carbon monoxide* (*DLCO*) measurements. CT is currently standard-of-care for lung imaging as it provides a high-resolution evaluation of lung parenchyma and surrounding pulmonary structures^12^. Spirometry is considered to be the gold-standard for accurate and repeatable measurements of lung function. Spirometric abnormalities, as well as other PFT results, are often required in the diagnosis of various lung diseases^13^. The association between quantitative CT imaging and PFT measurements is therefore highly relevant.

The identification of robust imaging biomarkers associated with pulmonary function may help standardize diagnostic criteria for lung disease^14^. This relationship has been widely studied to better characterize relevant pulmonary conditions such as, emphysema^15,16^, asthma^17^, interstitial lung disease^18^, lung inflammation^19^, chronic obstructive pulmonary disease^20,21^, and pulmonary fibrosis^22^. A majority of these approaches are based on texture analysis and intensity thresholding. We note that texture analysis is only one component of a typical radiomics pipeline. In addition to texture features, radiomic analyses often incorporate aspects of intensity^1–11^, morphology^1–11^, fractal geometry^23^, and higher-order features (e.g., from wavelet transformations^2^). Interactions between these different types of information may lead to novel insights and better characterization of the lungs in CT images.

A quantitative radiomics approach therefore has the potential to better characterize both anatomical and functional aspects of the lungs in CT images. However, the ability to draw meaningful conclusions from hyper-dimensional imaging data is often non-trivial^24^. To achieve our objective, therefore, we took an exploratory approach based on both univariate statistical analysis and dynamic data clustering. Dynamic clustering algorithms have gained considerable interest in recent years^25–31^, largely due to their unique capability to investigate *how* data clusters are formed. As such, dynamic frameworks provide an intuitive mechanism to better understand cluster formation in data with many degrees-of-freedom, and promote exploratory data analysis of highly complex datasets.

## Methods

The following methods were carried out in accordance with relevant guidelines and regulations. Retrospective data collection and analysis was completed with approval from the Duke University Health System Institutional Review Board. The study ID is Pro00067169; data collection was approved under a Waiver of Consent and HIPAA Authorization and a Notification of Decedent Research.

### CT image acquisition and lung segmentation

Sixty-four non-small cell lung cancer (NSCLC) patients were retrospectively identified and included in this study. For each patient, pre-treatment x-ray CT images were acquired under free-breathing conditions. Images were acquired on either a GE Lightspeed CT scanner (160 slices, 0.8 mm in plane resolution, 2.5 mm slice thickness, 120 kVp), or a Siemens Biograph mCT scanner (160 slices, 0.8 mm in plane resolution, 2 mm slice thickness, 120 kVp), and reconstructed using a filtered back projection reconstruction algorithm. Both scanners were calibrated using an identical QA protocol and phantom. The total lung volume was segmented from the CT images using commercially available contouring software, and the dynamic range of the image within the lungs was re-binned from 0-to-64 gray levels.

### Pulmonary function tests

Pulmonary function was quantified based on pre-treatment PFT results obtained within 6 weeks of image acquisition. Two common PFT metrics were investigated: (1) DLCO, and (2) *FEV*_1_. DLCO is a measurement of alveolar gas exchange to hemoglobin within the pulmonary capillaries. Low DLCO values are often associated with pulmonary arterial hypertension, emphysema, anaemia, and interstitial lung disease^32^. FEV_1_ measures gas flow within the lungs via spirometry, and is generally used in the clinical diagnosis of airway diseases. Low FEV_1_ values have been shown to be correlated with obstructive lung diseases such as emphysema, asthma, and COPD, among other respiratory conditions^33^. We considered both the *absolute* FEV_1_ (measured in liters/second) and the *percent predicted* FEV_1_ (FEV_1%_, an FEV_1_ measurement adjusted for co-linear biometric parameters, including age, sex, height, and ethnicity). For the latter, we also analyzed the data according to the diagnostic criteria proposed by the Global Initiative for Chronic Obstructive Lung Disease (GOLD)^34^.

### Radiomic feature extraction and pulmonary feature-space design

A total of 39 radiomic features were extracted from the total lung volume as potential biomarkers for pulmonary function. We define the resulting pulmonary radiomic feature space as a matrix,

1 $${\boldsymbol{ {\mathcal F} }}=({f}_{i,j})\in {{\mathbb{R}}}^{39\times 64}$$

where, columns denote patient-specific feature vectors, and rows denote feature measurements spanning the patient cohort. The (*i*, *j*)^*th*^ coordinate of ${\boldsymbol{ {\mathcal F} }}$ represents the measured value of the *i*^*th*^ radiomic feature as observed within the image of the *j*^*th*^ patient’s lungs. For simplicity, we denote the *j*^*th*^ patient’s radiomic feature vector as,

2 $$\begin{array}{ll}{{\bf{f}}}_{j}={({f}_{i,j}\,)}_{i=1}^{39}\in {{\mathbb{R}}}^{39}, & j=1,2,\ldots ,\,64.\end{array}$$

**Figure 1:**
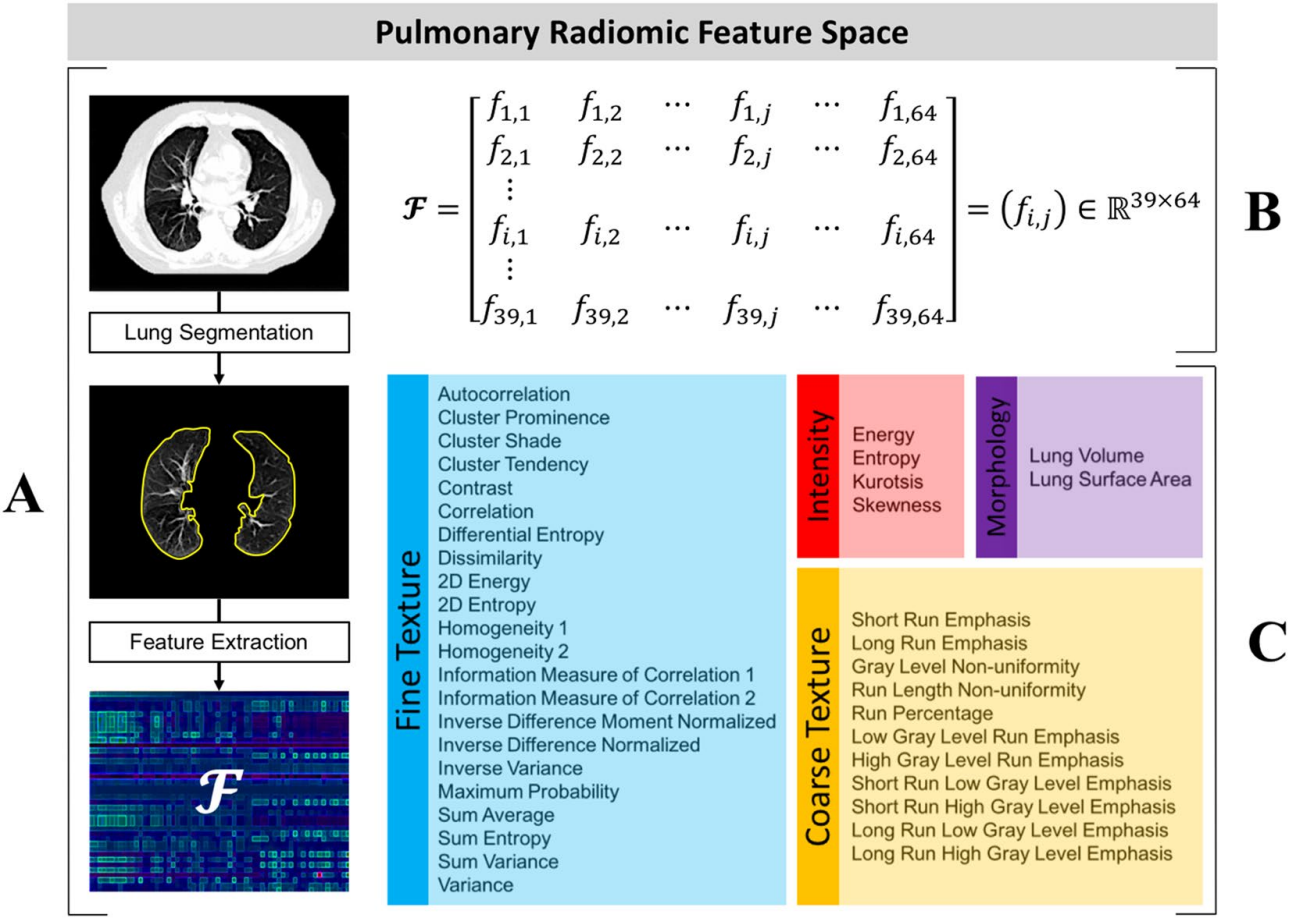
Pulmonary radiomic feature space design. (**A**) Radiomic features are extracted from CT-segmented lungs to derive a feature space, ${\boldsymbol{ {\mathcal F} }}$. (**B**) ${\boldsymbol{ {\mathcal F} }}$ is mathematically defined as a (39 x 64)-dimensional matrix. The ${(i,j)}^{th}$ coordinate of ${\boldsymbol{ {\mathcal F} }}$ represents the measured value of the *i*^*th*^ feature as observed within the image of the *j*^*th*^ patient. (**C**) The features used to construct ${\boldsymbol{ {\mathcal F} }}$, color coded by class.

A complete list of all 39 features that were used to construct Eq. (2) is shown in Fig. 1. These features were chosen to collectively capture the overall lung density, lung fine texture, lung coarse texture, and lung morphology. Each of these aspects represents a unique subset of ${\boldsymbol{ {\mathcal F} }}$:

- **Intensity** $({f}_{1,j},\ldots ,{f}_{4,j}\,)$: Features that measure various density characteristics of the lungs. These features were derived from the image’s gray-level histogram^2^.
- **Fine Texture** $({f}_{5,j},\ldots ,{f}_{26,j}\,)$: Features that measure the heterogeneity present within the detailed, high-resolution lung structure. These features were derived from the image’s Gray-Level Co-occurrence Matrix^35^.
- **Coarse Texture** $({f}_{27,j},\ldots ,{f}_{37,j}\,)$: Features that measure the heterogeneity present within the approximate, low-resolution lung structure. These features were derived from the image’s Gray-Level Run Length Matrix^36^.
- **Morphology** $({f}_{38,j},{f}_{39,j}\,)$: Lung surface area and volume^2^.

### Univariate association of lung radiomics data with pulmonary function

The univariate statistical association between the radiomics lung data and the PFT data was quantified as follows. The feature space, ${\boldsymbol{ {\mathcal F} }}$, was normalized using a z-score transformation. Each feature (i.e., each row vector of ${\boldsymbol{ {\mathcal F} }}$) was tested for normality using the Kolmogorov-Smirnov method^37^. Any feature that rejected the null hypothesis of a normal distribution was discarded from further univariate analysis. Pearson correlation coefficients were calculated between each of the remaining features and both *FEV*_1_ and *DLCO*. All *p*-values were adjusted for multiple hypotheses testing based on the false discovery rate procedure proposed by Benjamini *et al*.^38^, and *p* ≤ 0.05 was considered to be statistically significant.

### Multivariate association of lung radiomics data with pulmonary function

The multivariate association between the pulmonary radiomics data and the PFT data was analyzed using an unsupervised dynamic clustering algorithm called Langevin Annealing. A complete derivation and comprehensive validation of this technique is available in^31^. By modeling data as a dynamical system in equilibrium with a heat bath, Langevin Annealing uses physics intuition to find intrinsic structure within data. The Langevin dynamics of individual radiomic feature vectors lead to different metastable states, which are interpreted as clusters. As the convergence of many points to a common location is a clear and intuitive indication of clustered data, the underlying hypothesis here is that radiomic feature vectors which travel to similar locations of the phase-space should have similar underlying pulmonary phenotypes. Briefly, the clustering technique was implemented as follows.

#### Singular value decomposition

First, a singular value decomposition (SVD) was performed on the feature space,

3 $${\boldsymbol{ {\mathcal F} }}=\mathop{\sum }\limits_{i=1}^{39}{\sigma }_{i}{{\boldsymbol{u}}}_{i}{{{\boldsymbol{v}}}_{i}}^{T}$$

where, ${{\boldsymbol{u}}}_{i}$ and ${{\boldsymbol{v}}}_{i}$ are the left-and-right singular vectors of ${\boldsymbol{ {\mathcal F} }}$, and *σ*_*i*_ are the singular values of ${\boldsymbol{ {\mathcal F} }}$. Equation (3) was truncated to its 5 leading terms (90% of the variance in ${\boldsymbol{ {\mathcal F} }}$) to define a lower-dimensional feature space in the uncorrelated orthogonal basis, ${\{{{\boldsymbol{u}}}_{i}\}}_{i=1}^{5}$.

#### Formulation of a potential function, V(x)

A radial basis function (RBF) was then used to construct a probability density function of the feature space variable ${\boldsymbol{x}}\in {{\mathbb{R}}}^{5}$,

4 $$\psi ({\boldsymbol{x}})=\mathop{\sum }\limits_{j=1}^{64}-\frac{{|{\boldsymbol{x}}-{{\boldsymbol{u}}}_{j}|}^{2}}{2{\sigma }^{2}}$$

where, *σ* is the resolution of the chosen RBF kernel and ${{\boldsymbol{u}}}_{j}\in {{\mathbb{R}}}^{5}$ is the *j*^*th*^ patient’s radiomic feature vector defined by Eq. (2) in the truncated SVD space defined by Eq. (3). Using the approach proposed by Horn *et al*.^39^, $\psi ({\boldsymbol{x}})$ was then interpreted as the ground-state solution associated with the quantum mechanical Hamiltonian operator,

5 $$ {\mathcal H} \psi ({\boldsymbol{x}})=-\frac{{\varepsilon }^{2}}{2}{\nabla }^{2}\psi ({\boldsymbol{x}})+V({\boldsymbol{x}})\psi ({\boldsymbol{x}}),$$

to inversely generate the following potential function up to a constant,

6 $$V({\boldsymbol{x}})=\frac{\frac{{\varepsilon }^{2}}{2}{\nabla }^{2}\psi ({\boldsymbol{x}})}{\psi ({\boldsymbol{x}})}.$$

In Eqs (5) and (6), *ε* = *σ*^2^ is known as the semi-classical parameter, which is reminiscent of the rescaled, dimensionless Planck constant^31^. The resolution parameter, *σ*, was heuristically varied to construct different potential functions at various scales according to Eq. (6). At each scale, the potential landscape was visually inspected until a reasonably smooth function was identified that approximated the density of the radiomics data.

#### Langevin dynamics on V(x)

Given *V*(*x*), Langevin dynamics were formulated as the following stochastic differential equations of motion,

7 $$d{{\boldsymbol{u}}}_{j}={{\bf{p}}}_{j}dt$$

and

8 $$d{{\bf{p}}}_{j}=-\,\nabla V({{\boldsymbol{u}}}_{j})dt-\gamma {{\bf{p}}}_{j}dt+\sqrt{2\gamma {k}_{b}T}dB$$

where, ${{\bf{p}}}_{j}\in {{\mathbb{R}}}^{5}$ is a time-dependent momentum vector associated with the *j*^*th*^ patient, $B(t)\in {{\mathbb{R}}}^{5}$ is Brownian motion, *γ* is a damping coefficient, *k*_*b*_ is the Boltzmann constant, *T* is the temperature of the system, and $T < \frac{1}{2}{\sigma }^{2}$ defines the critical temperature relative to the resolution of the RBF kernel^31^. To preserve unit dimensions, we let *γ* = 1 and chose to work at the characteristic scale where *k*_*b*_ = 1. Detailed parameterization of Eqs (7) and (8) are described in^31^.

As the system evolves in time according to Eqs (7) and (8) it *dissipates* kinetic energy, which is balanced with corresponding *fluctuations* in the form of Brownian motion. As such, the time dynamics of individual feature vectors lead to different metastable states, which are interpreted as cluster centers. Clustering is achieved when subsets of the radiomics data aggregate near different potential minima. Langevin annealing offers some unique advantages. While radiomic feature vectors are pushed towards minima by the potential gradient, Brownian motion allows them to effectively tunnel through local potential barriers and escape saddle points into locations of the potential surface otherwise forbidden^31^. As a result, nearly degenerate local potential minima are able to merge into single clusters. Practically speaking, this allows for a high-resolution RBF kernel to be used, while still maintaining a narrow impulse response. The PFT data between different clusters was ultimately compared using an analysis of variance (ANOVA) approach and *p *≤ 0.05 was considered to be statistically significant.

## Results

### Univariate association of lung radiomics data with pulmonary function

Univariate results are summarized in Table 1. *DLCO* was found to be correlated with two coarse texture features, the strongest being *Short Run Low Gray Level Run Emphasis* (*ρ *= −0.53). FEV_1_ was found to be correlated with 24 different radiomic features. In particular, the coarse texture feature, *Long Run High Gray Level Emphasis*, demonstrated the strongest correlation (*ρ *= 0.72). Regarding lung intensity, fine texture, and morphology, the strongest correlations with FEV_1_ were *Skewness (ρ *= −0.62), *Sum Average (ρ *= 0.63), and *Surface Area (ρ* = −0.45), respectively.

**Table 1:** Univariate Correlation of Radiomics Data with Pulmonary Function Tests.

Feature	Correlation Coefficient	p-value
**Correlation of Coarse Texture with** ***DLCO***		
*Low Gray Level Run Emphasis*	*ρ* = −0.51	p = 0.011
*Short Run Low Gray Level Emphasis*	*ρ* = −0.53	p = 0.008
**Correlation of Intensity with** ***FEV***_***1***_		
*Energy*	*ρ* = 0.56	p < 0.001
*Entropy*	*ρ* = −0.46	p = 0.002
*Kurtosis*	*ρ* = −0.54	p < 0.001
*Skewness*	*ρ* = −0.62	p < 0.001
**Correlation of Fine Texture with** ***FEV***_***1***_		
*Autocorrelation*	*ρ* = 0.57	p < 0.001
*Cluster Prominence*	*ρ* = 0.34	p = 0.026
*Cluster Shade*	*ρ* = 0.45	p = 0.002
*Cluster Tendency*	*ρ* = 0.57	p < 0.001
*Correlation*	*ρ* = 0.55	p < 0.001
*2D Entropy*	*ρ* = 0.34	p = 0.025
*Information Measure of Correlation 2*	*ρ* = 0.35	p = 0.019
*Maximum Probability*	*ρ* = −0.42	p = 0.004
*Sum Average*	*ρ* = 0.63	p < 0.001
*Sum Entropy*	*ρ* = 0.39	p = 0.009
*Sum Variance*	*ρ* = 0.56	p < 0.001
*Variance*	*ρ* = 0.54	p < 0.001
**Correlation of Coarse Texture with** ***FEV***_***1***_		
*Gray Level Non-uniformity*	*ρ* = −0.36	p = 0.018
*Low Gray Level Run Emphasis*	*ρ* = −0.54	p < 0.001
*High Gray Level Run Emphasis*	*ρ* = 0.47	p = 0.001
*Short Run Low Gray Level Emphasis*	*ρ* = −0.56	p < 0.001
*Short Run High Gray Level Emphasis*	*ρ* = 0.40	p = 0.007
*Long Run High Gray Level Emphasis*	*ρ* = 0.72	p < 0.001
**Correlation of Morphology with FEV**_**1**_		
*Surface Area*	*ρ* = −0.45	p = 0.002
*Volume*	*ρ* = −0.42	p = 0.006

### Multivariate association of lung radiomics data with pulmonary function

**Figure 2:**
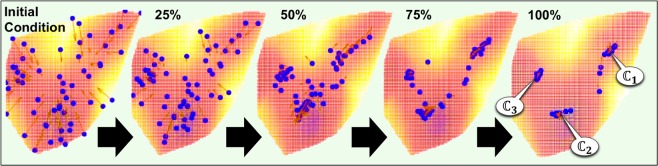
Dynamic clustering of pulmonary radiomics data with Langevin Annealing. The location of each patient’s radiomic vector is plotted in blue, relative to a projection of the potential surface generated from the radiomics data at σ = 0.08. Five sequential time frames are included to demonstrate the dynamic clustering process, which results in 3 distinct clusters (${{\mathbb{C}}}_{1}$, ${{\mathbb{C}}}_{2}$, ${{\mathbb{C}}}_{3}$). The red arrows represent the Langevin net-force that each radiomic vector is subject to as a function of space and time.

Clustering of the radiomics data according to Langevin Annealing is demonstrated in Fig. 2. The blue data points represent the location of each patient’s radiomic feature vector as a function of computation time (i.e., *t* = 0 to *t* = 1, with a time-step of *δt* = 0.01), relative to a projection of the potential function, *V*(***x***). In addition, the red arrows represent the Langevin net-force that each feature vector is subjected to according to Eqs (6) and (7). The location of each data point at time *t* = 0 was used to construct the potential function at resolution, $\sigma =0.08$, according to Eq. (6). When *σ* = 0.08, the radiomic feature space’s corresponding potential landscape exhibited three distinct minima. As demonstrated in Fig. 2, three unique patient clusters were identified within the radiomics data at these minima.

**Figure 3:**
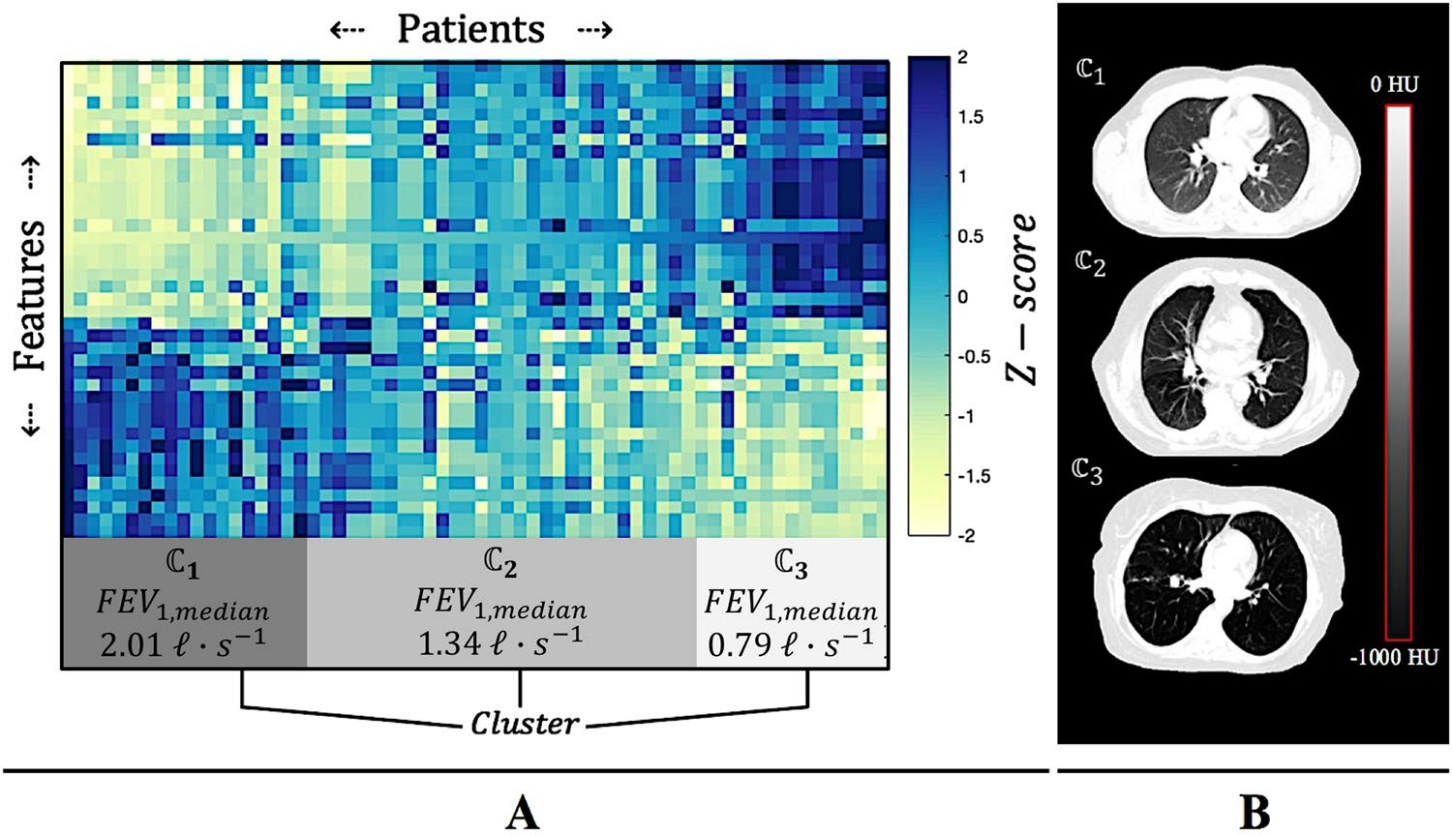
Bi-clustering of the Euclidean radiomic feature space. (**A**) The radiomic feature space is represented as a matrix, ${\boldsymbol{ {\mathcal F} }}$, where column vectors denote the radiomic feature vectors of each patient, and row vectors denote a particular feature measurement made across the patient cohort. The feature space has been sorted according to the 3 unique clusters (${{\mathbb{C}}}_{1}$, ${{\mathbb{C}}}_{2}$, ${{\mathbb{C}}}_{3}$) found via Langevin Annealing (Fig. 2). Each cluster corresponds to a statistically significant median *FEV*_*1*_ value, indicating that patients with similar radiomic lung signatures also presented with similar spirometry measurements. (**B**) Illustrating examples of CT images taken from each of the three identified clusters.

Figure 3, shows ${\boldsymbol{ {\mathcal F} }}$ mapped back to its original Euclidean coordinate system, where the data has been sorted according to the clusters found via the Langevin dynamics in the Hilbert Space shown in Fig. 2. The intensity value of the (*i*, *j*)^*th*^ element of ${\boldsymbol{ {\mathcal F} }}$ signifies the z-score of the *i*^*th*^ feature as measured within the image of the *j*^*th*^ patient. Columns of ${\boldsymbol{ {\mathcal F} }}$ denote the measured radiomic feature vectors of each patient as defined via Eq (2). Row vectors of ${\boldsymbol{ {\mathcal F} }}$ denote individual radiomic features measured across the patient cohort, as defined in Fig. 1.

Each cluster in this 39-dimensional space corresponds to a statistically significant median *FEV*_1_ value, indicating that patients with similar lung radiomics data also presented with similar spirometry measurements. Specifically, clusters 1, 2 and 3 had median *FEV*_1_ values of 2.01 $\ell {s}^{-1}$, 1.34 $\ell {s}^{-1}$, and 0.79 $\ell {s}^{-1}$, respectively. These clusters were found to be statistically significant according to ANOVA results (p = 2.3E-6). When considering the *FEV*_1_ *percent predicted*, *FEV*_1*%*_, median values of each cluster were 66%, 49.0%, and 32.5%, respectively, and were also statistically significant (p = 2.5E-4), indicating that co-linear biometric parameters did not affect the clustering analysis. Table 2 summarizes clustering results as interpreted by the Global Initiative for Chronic Obstructive Lung Disease (GOLD)^34^, where the percentage of patients in each cluster are categorized into different diagnostic spirometry thresholds.

**Table 2:** Cluster-by-cluster breakdown of *FEV*_*1%*_ values according to the GOLD standard.

Percentage of Predicted FEV1 Value	Percentage of patients in ${{\mathbb{C}}}_{1}$	Percentage of patients in ${{\mathbb{C}}}_{2}$	Percentage of patients in ${{\mathbb{C}}}_{3}$
*Mild* (80% or above)	18%	6%	0%
*Moderate* (50–79%)	55%	39%	16%
*Severe* (30–49%)	27%	44%	42%
*Very Severe* (29% or less)	0%	11%	42%

## Discussion

Novel radiomic applications are highly desired as potential avenues to personalized medicine. This exploratory radiomics study demonstrated a relationship between quantitative imaging features on CT and pulmonary function. In particular, our results indicate that high-throughput radiomics data extracted from the lungs may be associated with pulmonary function as measured by common PFT metrics. This finding is supported by both univariate and multivariate analyses. Overall, patients with larger lungs of homogeneous, low attenuating pulmonary tissue were found to have lower *FEV*_1_, and patients with dense, heterogeneous pulmonary tissue were found to have higher *DLCO*.

Univariate analysis identified *FEV*_*1*_ to be independently correlated with several radiomic features. In particular, the strongest correlation from each feature class was *Skewness* (*ρ* = −0.62), *Sum Average* (*ρ* = 0.63), *Long Run High Gray Level Emphasis (ρ* = 0.72), and *Surface Area (ρ* = −0.45). Collectively, these results suggest that patients with larger lungs of homogeneous, low density pulmonary tissue often presented with worse *FEV*_1_ values, relative to patients with smaller lungs of heterogeneous, dense pulmonary tissue.

*Long Run High Gray Level Emphasis* (*LRHGLE*) is a joint-measure of image intensity and image homogeneity. This feature therefore quantifies the joint-relationship between CT attenuation and HU homogeneity. A high *LRHGLE* value indicates dense, homogenous tissue, while a low value indicates low attenuating, heterogeneous tissue. In this study, the observed direct relationship between *LRHGLE* and *FEV*_1_ implies that: (a) patients with better spirometry also presented with dense, homogenous lungs; and (b) patients with worse spirometry also presented with low attenuating, heterogeneous lungs.

Two coarse texture features were found to be inversely correlated with *DLCO*: *Low Gray Level Run Emphasis* (*ρ* = −0.51) and *Short Run Low Gray Level Emphasis* (*ρ* = −0.53). *Low Gray Level Run Emphasis* (LGLRE) is a measure of CT attenuation only, while *Short Run Low Gray Level Emphasis* (*SRLGLE*) is a joint-measure of both CT attenuation and HU heterogeneity. The stronger correlation coefficient of *SRLGLE* suggests that spatially-encoded texture information may potentially be more important than intensity information in isolation. This conclusion remains limited, however, and should be tested on a more comprehensive sample data set with a larger sample size. By definition, a high *SRLGLE* value indicates low attenuating, heterogeneous tissue. As *SRLGLE* is an antagonistic feature to *LRHGLE*, the negative correlation coefficient here implies these findings are qualitatively consistent with the *FEV*_1_ results discussed above. That is, the observed inverse relationship between *SRLGLE* and *DLCO* implies that (a) patients with better diffusing capacity also presented with dense, homogenous lungs, and (b) patients with worse diffusing capacity also presented with low attenuating, heterogeneous lungs.

It is reasonable to hypothesize that reduced lung function is associated with a loss of healthy pulmonary tissue being replaced by an increased amount of air in the lungs in patients with emphysema^40^. An increase in air would result in a reduction of CT attenuation coefficients, which in turn would result in (a) lower *LRHGLE* values, and (b) higher SRLGLE values. These findings are consistent with other studies that have used CT lung density metrics in the diagnosis and characterization of emphysema^15^, asthma^17^, lung inflammation^19^, COPD^20^, and cystic fibrosis^41^. In particular, Barjaktarevic *et al*. found a positive correlation between *DLCO* and the *total tissue volume* measured in CT images. While our conclusions are in agreement, we found *SRLGLE* to be more strongly correlated with DLCO than the results reported by Barjaktarevic *et al*.^42^ for *total tissue volume* (*ρ* = −0.530 vs. *ρ* = 0.498). *Total tissue volume* is a first-order statistical feature measured via intensity thresholding with often arbitrary cutoffs. Unlike *SRLGLE*, *total tissue volume* therefore does not capture spatially-encoded information (i.e., texture).

Our results provide evidence that lung texture – in addition to lung density – is partly responsible for the association between pulmonary function and CT imaging. This is significant, as first-order features extracted from image histograms may not be sufficient in the presence of complex, mixed pathologic processes^43^. In fact, CT texture analysis of the lungs has been applied to interstitial lung disease diagnosis^18^, emphysema quantification^16^, lung abnormality detection (e.g., honeycombing, ground glass, etc.)^44^, pulmonary thromboembolism characterization^45^, pulmonary fibrosis^22^, and COPD^21^.

As stated in the introduction, texture analysis is just one component of a typical high-throughput radiomics pipeline. In addition to texture features, radiomic analyses often incorporate aspects of intensity, morphology, fractal geometry, and higher-order feature extraction. Interactions between these different types of information may lead to novel insights and better characterization of the lungs in CT images. Our multivariate clustering results support this notion.

A significant association was found between the clustered radiomics data and *FEV*_1_, indicating that patients with similar radiomic vectors also presented with comparable spirometry performance. Further, a significant association was found between the clustered radiomics data and *FEV*_1*%*_, indicating that well-known biometric collinearities had a nominal effect on our clustering results. Our cluster-by-cluster analysis using the guidelines developed by the Global Initiative for Chronic Obstructive Lung Disease (GOLD) further strengthened these findings. In particular, 84% of patients in cluster 3 were identified with either *Severe* or *Very Severe* pulmonary obstructions, compared to only 56% of patients in cluster 2, and 27% of patients in cluster 1. Likewise, 73% of the patients in cluster 1 were identified with either Mild or Moderate pulmonary obstructions, compared to only 45% of patients in cluster 2, and 16% of patients in cluster 3.

While this study presents a novel approach to quantifying pulmonary function on CT, it has several limitations. First, our analysis was performed in the context of a retrospective study, on a relatively small cohort of early-stage lung cancer patients. Generalization of our findings to patients with other respiratory conditions remains unclear, and warrants future investigation. Further, our analysis was performed on imaging data that was acquired under free-breathing conditions. While this is a standard acquisition technique in radiation oncology departments, generalization of our methodology to other acquisition techniques also needs further study. Breath-hold images, which would maximize radiomics data fidelity, may not always be a standard-of-care option for lung cancer patients^3^. Therefore, characterization of radiomics analysis across different imaging techniques is important. Finally, it is generally known that radiomic features are highly sensitive to acquisition parameters, often leading to non-reproducible imaging features^3,46,47^. In our study, we attempted to account for this effect by using only free-breathing images with nominal differences in acquisition parameters. We did not find any association between the pulmonary radiomics data and the CT scanner unit, which is consistent with results from^3^. Nonetheless, the standardization of radiomic features is an essential aspect to future clinical implementation.

In conclusion, this paper has presented an exploratory investigation of the relationship between high-throughput CT radiomics data and pulmonary function. A quantitative radiomics approach has the potential to objectively measure changes in lung function based on routine CT imaging. If identified, robust imaging biomarkers associated with pulmonary function may lead to improved quantification and standardization of diagnostic criteria for different lung diseases. Such radiomic features would ultimately serve in a complimentary role to conventional diagnostic criteria, clinical findings, and expert observations. As such, pulmonary radiomic analysis may eventually lead to clinically useful tools that can help to improve diagnosis, prognosis, and patient care.

## Data Availability

The datasets generated during and/or analyzed during the current study are available from the corresponding author on reasonable request.
